# Addressing Errors in Scientific Publishing: The Role of Errata

**DOI:** 10.31729/jnma.8759

**Published:** 2024-09-30

**Authors:** Sushant Regmi

**Affiliations:** 1 Managing Editor, Journal of Nepal Medical Association, Exhibition road, Kathmandu, Nepal

Clinical evidence from systematic research is one of the foundations of the evidence as highlighted by David Sackett.^1^ In the current practice of evidence-based medicine, disseminating accurate and reliable information through medical research is of utmost importance. A manuscript undergoes multiple editorial steps and a rigorous peer review process pre-production; however, errors still occur in scientific publication and are encountered post-production.

The National Library of Medicine (NLM) considers partial retractions, corrigenda, corrections, and addenda all as errata.^2^ Errata is published when there are errors encountered post-publication. During the publication process errors can occur as methodical errors, typographical mistakes, misinterpretation of data, and miscalculations. These errors regardless of their nature can affect the results and mislead readers. In worst cases, these can influence clinical practice based on flawed evidence. Therefore, it is necessary to address these errors as soon as possible and transparently.

When an error is identified post-publication, journals have a responsibility to correct the record. The publication of an erratum is a standard practice in scholarly publishing to amend such mistakes.^3^ Correcting the record and publication of erratum is a crucial step for several reasons accuracy, accountability, and transparency. Journal of Nepal Medical Association (NMA) too is open to correcting the record and publication of erratum in maintaining accuracy, accountability, and transparency and does so abiding by the International Committee of Medical Journal Editors (ICMJE) and Committee on Publication Ethics (COPE) guidelines. ICMJE emphasizes that corrections should be made following the guidelines developed by COPE promptly detailing and citing all the changes from the original publication and be made accessible to ensure that readers have access to the most accurate information available.^4^

## IMPACT OF ERRATA

Recognizing and addressing mistakes is important in maintaining the integrity of scientific research. By publishing an erratum, publishers can ensure scientific accuracy and maintain ethical standards. This practice has several beneficial impacts:

**1. Enhancing Trust and Transparency**

Trust is the cornerstone of scientific research. When journals openly admit and correct errors, it fosters trust among researchers, clinicians, and the public. Journal editors are sometimes reluctant to publish corrections or write an erratum.^5^ But to preserve public trust and integrity of a journal it is essential to provide correct information to the readers. Moreover, it reassures readers that the journal is committed to the highest standards of accuracy and transparency.

**2. Encouraging Ethical Conduct**

Ethical conduct is the foundation for any scientific research. By correcting the errors journals are also ensuring ethical scientific practice. Findings from 208 cases of research misconduct from the US Office of Research Integrity, spanning from 1992 to 2011 revealed that retractions and corrections are increasingly addressing ethical issues and becoming more transparent.^6^ This trend likely reflects a journals are open to admitting to errors and want to ensure ethical practice in scientific publishing.

**3. Mitigating the Impact of Errors**

Errors can negatively impact the scientific community. The impact might be ranging but not limited to misinterpretations, deriving false conclusions, and influencing harmful clinical practice. Research with errors does not derive knowledge that is useful for the scientific community and all the resources that were used to conduct the study will be wasted. By addressing these errors, journals help prevent the propagation of misinformation and eventually influence good clinical practice.

While errata are crucial for correcting post-publication errors, it is equally important to minimize these errors through robust peer review and editorial processes. However, the process of issuing errata is not without challenges. Identification of errors stands out as one of the major challenges in addition to the author not wanting to admit to their mistakes in fear of damaging their reputation.

JNMA has a pre-production checklist and postproduction checklist in place to mitigate errors during publishing. Errors, when encountered in this journal, are when the published manuscript again undergoes screening through the post-publication checklist and sometimes encountered as a letter to the editor. Regardless of the source, appropriate measures are taken based on existing guidelines.
